# Lung lobe segmentation: performance of open-source MOOSE, TotalSegmentator, and LungMask models compared to a local in-house model

**DOI:** 10.1186/s41747-025-00623-9

**Published:** 2025-09-04

**Authors:** Elaheh Amini, Ran Klein

**Affiliations:** 1 Systems and Computer Engineering, Carleton University, Ottawa, ON Canada; 2 Division of Nuclear Medicine and Molecular Imaging, Faculty of Medicine, University of Ottawa, Ottawa, ON Canada; 3 Department of Nuclear Medicine and Molecular Imaging, The Ottawa Hospital, Ottawa, ON Canada

**Keywords:** Data variability, Deep learning, Image processing (segmentation), Lung, Tomography (x-ray computed)

## Abstract

**Background:**

Lung lobe segmentation is required to assess lobar function with nuclear imaging before surgical interventions. We evaluated the performance of open-source deep learning-based lung lobe segmentation tools, compared to a similar nnU-Net model trained on a smaller but more representative clinical dataset.

**Materials and methods:**

We collated and semi-automatically segmented an internal dataset of 164 computed tomography scans and classified them for task difficulty as easy, moderate, or hard. The performance of three open-source models—multi-organ objective segmentation (MOOSE), TotalSegmentator, and LungMask—was assessed using Dice similarity coefficient (DSC), robust Hausdorff distance (rHd95), and normalized surface distance (NSD). Additionally, we trained, validated, and tested an nnU-Net model using our local dataset and compared its performance with that of the other software on the test subset. All models were evaluated for generalizability using an external competition (LOLA11, *n* = 55).

**Results:**

TotalSegmentator outperformed MOOSE in DSC and NSD across all difficulty levels (*p* < 0.001), but not in rHd95 (*p* = 1.000). MOOSE and TotalSegmentator surpassed LungMask across metrics and difficulty classes (*p* < 0.001). Our model exceeded all other models on the internal dataset (*n* = 33) in all metrics, across all difficulty classes (*p* < 0.001), and on the external dataset. Missing lobes were correctly identified only by our model and LungMask in 3 and 1 of 7 cases, respectively.

**Conclusion:**

Open-source segmentation tools perform well in straightforward cases but struggle in unfamiliar, complex cases. Training on diverse, specialized datasets can improve generalizability, emphasizing representative data over sheer quantity.

**Relevance statement:**

Training lung lobe segmentation models on a local variety of cases improves accuracy, thus enhancing presurgical planning, ventilation-perfusion analysis, and disease localization, potentially impacting treatment decisions and patient outcomes in respiratory and thoracic care.

**Key Points:**

- Deep learning models trained on non-specialized datasets struggle with complex lung anomalies, yet their real-world limitations are insufficiently assessed.
- Training an identical model on a smaller yet clinically diverse and representative cohort improved performance in challenging cases.
- Data diversity outweighs the quantity in deep learning-based segmentation models.
- Accurate lung lobe segmentation may enhance presurgical assessment of lung lobar ventilation and perfusion function, optimizing clinical decision-making and patient outcomes.

**Graphical Abstract:**

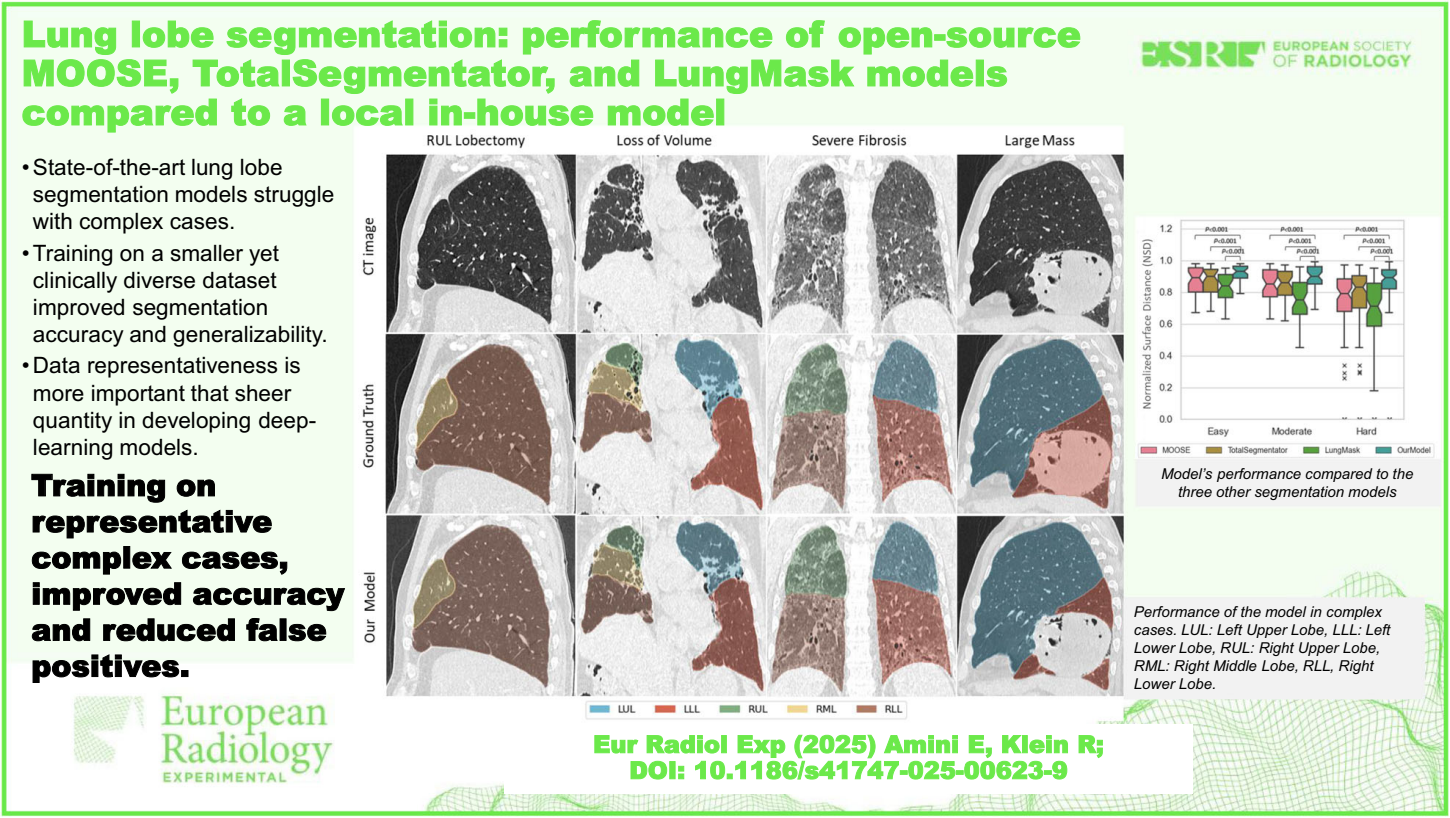

## Background

The human lungs are typically divided into five anatomically and physiologically isolated bronchopulmonary lobes, which are separated by three main pulmonary fissures [1]. This isolation confines certain diseases within specific lobes [2–4], enabling treatments such as lobectomy to preserve the function of unaffected regions. By quantifying the function of each individual lobe, the residual function may be estimated to aid treatment planning and prognostication. While whole lung perfusion and ventilation function measurements can be routinely performed with single-photon emission computed tomography (SPECT), a coregistered x-ray computed tomography (CT) is essential to segment the lungs into their lobes.

Although CT provides excellent contrast for lung tissue and fissures, segmentation performance is affected by incomplete or displaced fissures [5], the presence of abnormalities or diseases, imaging variability, and protocol differences [6]. Increasing imaging studies and workloads make manual or semiautomatic segmentation impractical, limiting routine lung lobe function quantification in clinical practice.

Early lung lobe segmentation approaches using classical image analysis methods relied on fissure detection, and their reliability was limited to high-quality CT and fissures with good visibility, integrity, and differentiability [7], and the absence of severe disease. Low-resolution scans or certain pathologies can compromise fissure detection due to their inherent assumptions about fissure intensity [8–10], orientation [10], shape [11, 12], and continuity, as well as lung parenchyma spatial uniformity. While some methods incorporated spatial data from neighboring structures, like airways and vessels [13, 14], abnormalities in these structures can also introduce errors [7]. Recent advances in machine learning-based segmentation models, including U-Net in 2015 [15] and later the nnU-Net algorithm [16], have produced a dramatic improvement in organ segmentation performance, including the lung lobes. Unlike classical methods, supervised machine-learning models are dependent on representative image data that have already been segmented to learn from. If the training data is not representative of the clinical data, their performance may be degraded when applied clinically.

In this study, we aimed to assess the performance of existing open-source software tools for CT-based lung lobe segmentation using our curated, clinically representative dataset. We benchmarked software performance with regard to rated task difficulty and studied the limitations of each software. Additionally, we sought to train our own segmentation model on local data to evaluate the impact of using clinically representative data on the performance of these models.

## Materials and methods

Briefly, this work describes the curation of CT images representative of lung lobe segmentation in our Nuclear Medicine clinic for the purpose of lung lobe function quantification. It then characterizes the performance of three open-source software programs for lung lobe segmentation, as well as an in-house developed software trained using our internal data. Finally, an independent, external, open challenge image set is further used to characterize the performance of all four software.

### Internal dataset

The database creation received approval from the Research Ethics Board (Protocol ID: 20220303-01H), making secondary use of clinical data. To develop a clinically representative dataset, we retrospectively collected recent chest CT scans of patients who had undergone lung perfusion SPECT for pre-operative lung function quantification prior to November 2023. To minimize radiation exposure and ensure quality, our practice uses prior diagnostic and low-dose CT images. Thus, the CTs in this database cover a broad range of chest diagnostic and low-dose scans acquired on various devices, with varying acquisition and reconstruction parameters, and with or without CT contrast.

#### Data preparation

We collated a dataset of 174 chest CT scans along with their corresponding clinical lung lobe annotations, excluding 10 atypical scans. These scans either could not be reliably segmented or, despite being reliably segmented, the severity and rarity of their abnormalities could affect the training process (see Fig. S1).

As our dataset covers a wide spectrum of pathologies, anatomies, imaging parameters, and fissure appearances, proper data stratification was required. To this end, clinical reports and imaging parameters for the CT studies were also collected. Each scan was assigned a segmentation difficulty score from three categories: easy, moderate, or hard. This scoring was based on visual assessment of the images in terms of fissure perceptibility and evaluation of segmentation results from both conventional and machine learning-based algorithms. Radiology reports were also consulted concerning the presence and severity of diseases (see Supplemental Material: Task Difficulty Assignment). To further characterize the clinical diversity of our dataset, we reviewed patient records and CT reports to document the underlying pulmonary conditions represented.

#### Ground truth segmentation

Following our clinical workflow, the latest chest CT for each patient was retrieved and semi-automatically segmented using Hybrid3D^TM^ software (Hermes Medical Solutions). All the segmentations were supervised by a medical physicist and a physician, both with more than 10 years of experience. However, this software can struggle with severe pathologies due to its reliance on thresholding and region-growing methods for segmenting the lungs. It may either fail to segment the lungs or omit the affected diseased (dense) areas. In such cases, we employed a pre-trained lung lobe segmentation algorithm, trained on part of our dataset, to obtain an initial segmentation, followed by manual corrections using the 3D Slicer software [17] for fixing any remaining inaccuracies.

#### Training and evaluation data preparation

A total of 164 CT scans with corresponding lung lobe segmentations resulted and were stratified by segmentation task difficulty to evaluate the performance of various lung lobe segmentation tools on an out-of-sample test set. Among these cases, 81 were classified as hard, 41 as moderate, and 42 as easy. Additionally, we employed this dataset to train a local nnU-Net model to assess the influence of dataset diversity on a model’s accuracy.

### Open-source models

We evaluated the performance of three open-source lung lobe segmentation algorithms: multi-organ objective segmentation (MOOSE) [18, 19], TotalSegmentator [20, 21], and Johof Lung Segmentation (LungMask) [22, 23]. Detailed descriptions of these algorithms and their respective training datasets can be found in the Supplemental material (Open-source software tools).

### Locally trained nnU-Net model

To examine the influence of dataset diversity on the performance of a deep learning model for lung lobe segmentation, we further trained an nnU-Net model using our curated dataset. We performed a manual stratification of the data based on segmentation difficulty to ensure effective model training. For training and validation, the dataset was split into 20% (33 cases; 8 easy, 9 moderate, and 16 hard) reserved as a test set, 15% (20 cases; 4 easy, 6 moderate, and 10 hard) used for validating the training process, and the remaining 65% (111 cases; 30 easy, 26 moderate, and 55 hard) for training. We used the 3D full-resolution nnU-Net configuration for training, as it empirically demonstrated the best performance on our dataset.

### Performance evaluation on the internal dataset

#### Standard lobar segmentation

For our evaluation, we used the Dice similarity coefficient (DSC), the 95th percentile Hausdorff distance (rHd95), and normalized surface distance (NSD) segmentation metrics [24, 25]. NSD is useful because it is bounded between 0 and 1, avoiding issues associated with metrics that can produce infinitely large values for studies with false-positive (FP) class predictions. Furthermore, it allows for an acceptable margin of deviation between predicted and ground truth boundaries, making it robust to minor inaccuracies of the ground truth annotations. We set a 2-mm tolerance for our evaluation, which reflects the expected level of variability that may occur if two different annotators were to delineate the lung and lobe boundaries. All three performance metrics were displayed as cumulative distribution functions (CDF) for simple visual comparison. For performance metrics that are ideally high (*e.g.*, DSC and NSD), a good response result has a long, low tail at 0 and an abrupt rise to 1 on the right. For performance metrics that are ideally low (*e.g.*, rHd), a good response shows a steep rise to 1 on the left, followed by a plateau. In cases with fewer than five lobes, models may be prone to predicting nonexistent lobes due to the underrepresentation of such cases in the training data, resulting in undefined rHd95 values for those lobes. These values were excluded on a per-lobe basis to ensure meaningful evaluation. All such scans were categorized as hard difficulty.

#### FP prediction (missing lobe cases)

We assessed the models’ handling of cases where the ground truth includes fewer than the usual five lobe classes (*i.e.*, prior lobectomy or collapsed lobe) using the class FP metric. FP was defined as the ratio of cases with erroneously segmented missing lobes to the number of images with missing lobes. FPs were stratified by missing lobe.

### Performance on the external dataset

To compare our results with previous lung lobe segmentation models, we used the lobe and lung analysis 2011 (LOLA11) challenge dataset [26] as our external dataset, which includes 55 pathologically diverse chest CT scans. The ground truth data (lung and lobe segmentation) are maintained private to prevent its use in model training, making LOLA11 ideal for benchmarking. We submitted the results from all four algorithms to the concluded LOLA11 competition with full acknowledgment given to the respective model developers. Segmentation results are evaluated using the Intersection over Union (IoU) metric.

### Computer environment

Segmentation models were trained and evaluated on a local workstation with an Intel Core i9 3.0 GHz CPU, 128 GB RAM, and an Nvidia GeForce RTX 3090 Ti GPU on Microsoft Windows 10. All models were developed using the PyTorch deep-learning library [27]. All analysis was performed in Python.

### Statistical analysis

To select the appropriate statistical analysis tool, we used the Kolmogorov–Smirnov test to assess the normality of our measurements for each evaluation metric across all lobes. To assess the statistical significance of performance differences between models and difficulty categories, we used the Wilcoxon signed rank and the Kruskal–Wallis tests, respectively. Regardless, the Bonferroni correction was applied in cases where multiple comparisons were made. If a significant difference was found between difficulty categories for an evaluation metric of a method, post-hoc pairwise comparisons were performed using the Mann–Whitney test to identify pairs responsible for the difference. The initial significance level was set at 0.05.

## Results

**Fig. 1:**
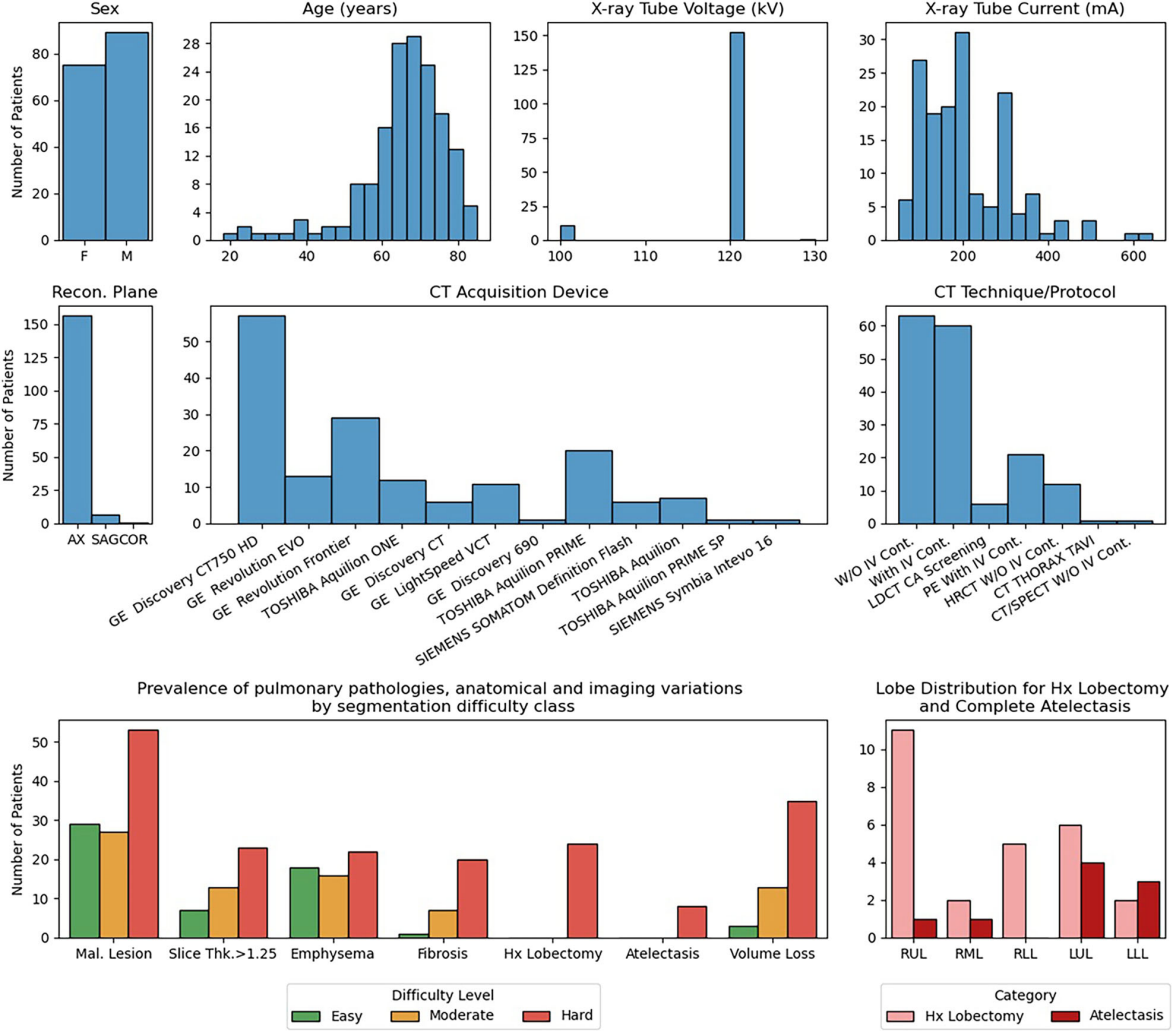
Distribution of imaging parameters of the CT studies, frequency of key pathologies, and imaging variations across three different segmentation difficulty classes, and distribution of missing lobe cases across lobes. AX, Axial; Cont., Contrast; COR, Coronal; Hx, History; LUL, Left upper lobe; LLL, Left lower lobe; Mal., Malignant; RML, Right middle lobe; RLL, Right lower lobe; RUL, Right upper lobe; SAG, Sagittal; Thk., Thickness; W, With; W/O, Without

Our internal database consisted of 164 images with complete lung lobe segmentations. A summary of patient demographics, image generation, and medical conditions of these studies is shown in Fig. 1. In addition, all CT matrix sizes were 512 × 512 pixels per axial slice with slice thickness ranging from 0.8 mm to 5.0 mm. Of these images, 42, 41, and 81 images were scored as easy, moderate, or hard difficulty, respectively.

Our dataset included the following conditions with varying severity, and often with overlapping presence in individual patients: 126 cases with malignant lesions, 7 cases with benign pulmonary lesions, 68 cases with emphysematous changes, 3 cases of pneumonia, 32 cases with fibrotic lung disease or post-treatment fibrosis, 9 cases with near-complete or complete lobar atelectasis, 26 postlobectomy cases, 61 cases with localized or global volume loss due to disease or treatment, and 26 patients referred for pre-lung transplant evaluation.

Notably, in 8 of the 164 included cases, the clinical segmentation software was unable to generate an initial lung segmentation due to extensive disease involvement. Five of these patients were awaiting lung transplantation, underscoring the complexity and severity of cases in our dataset.

### Open-source software evaluation

#### Quantitative performance

In evaluating MOOSE, TotalSegmentator, and LungMask on the internal dataset (*n* = 164), good performance was noted for TotalSegmentator and MOOSE in easy and moderate difficulty cases with median DSC ≥ 0.94, rHD95 ≤ 7.16, and NSD ≥ 0.87 (Table 1). All three metrics correlated with class difficulty (see Fig. S2). Specifically, the hard class exhibited significantly poorer (higher rHd95 and lower DSC and NSD) median performance (*p* < 0.001) compared to both the moderate and easy classes, while the moderate class also showed significantly poorer median performance (*p* < 0.05) than the easy class.

**Table 1:** Segmentation performance for open-source models on the entire internal dataset

*n* = 164	Easy (42)					Moderate (41)					Hard (81)				
	Mean	SD	Q1	Median	Q3	Mean	SD	Q1	Median	Q3	Mean	SD	Q1	Median	Q3
	**DSC**														
MOOSE	0.95	0.03	0.94	0.96	0.97	0.93	0.08	0.93	0.96	0.97	0.79	0.3	0.8	0.92	0.96
TotalSegmentator	**0.95**	**0.03**	**0.94**	**0.97**	**0.97**	**0.94**	**0.09**	**0.93**	**0.96**	**0.97**	**0.8**	**0.3**	**0.84**	**0.94**	**0.97**
LungMask	0.95	0.04	0.94	0.96	0.97	0.92	0.09	0.91	0.94	0.97	0.77	0.3	0.78	0.91	0.95
	**rHd95 (mm)**														
MOOSE	**7.48**	**7.03**	**2.68**	**4.94**	**10.27**	**10.96**	**11.81**	**3.05**	**7.14**	**13.41**	**19.21**	**21.69**	**5.14**	**11.3**	**21.63**
TotalSegmentator	**7.49**	**6.78**	**2.55**	**5.08**	**9.83**	**11.52**	**13.51**	**3.15**	**7.16**	**13.39**	**19.06**	**21.84**	**4.59**	**10.35**	**22.88**
LungMask	11.23	9.45	5	8.04	14.8	17.13	15.19	5.7	12.24	23.65	25.76	22.45	10.31	17.84	32.62
	**NSD**														
MOOSE	0.87	0.08	0.81	0.9	0.94	**0.85**	**0.11**	**0.79**	**0.87**	**0.93**	0.7	0.27	0.63	0.8	0.89
TotalSegmentator	**0.88**	**0.08**	**0.82**	**0.9**	**0.94**	**0.85**	**0.12**	**0.79**	**0.88**	**0.93**	**0.72**	**0.28**	**0.67**	**0.83**	**0.9**
LungMask	0.83	0.1	0.77	0.85	0.9	0.78	0.14	0.7	0.8	0.89	0.63	0.27	0.54	0.71	0.83

Results are combined across all lobes for each difficulty class. Bold values represent the highest performing models in each metric, difficulty class, and model category. Column header numbers in parentheses represent the number of images

Both MOOSE and TotalSegmentator showed statistically significantly better average performance (*p* < 0.01) across all difficulty classes for all evaluation metrics when compared to LungMask on the entire dataset. Furthermore, TotalSegmentator outperformed MOOSE across all difficulty classes for the DSC metric (*p* < 0.001) and for the easy and hard classes in NSD (*p* < 0.001). However, their performance by the rHd95 metric was similar across all difficulty levels (*p* = 1), suggesting outlier surface voxels with both models. Regardless, when considering the overall predicted volume, accounted for by DSC and NSD metrics, TotalSegmentator demonstrates statistically significantly better performance than MOOSE, but the magnitude of these differences was small.

**Fig. 2:**
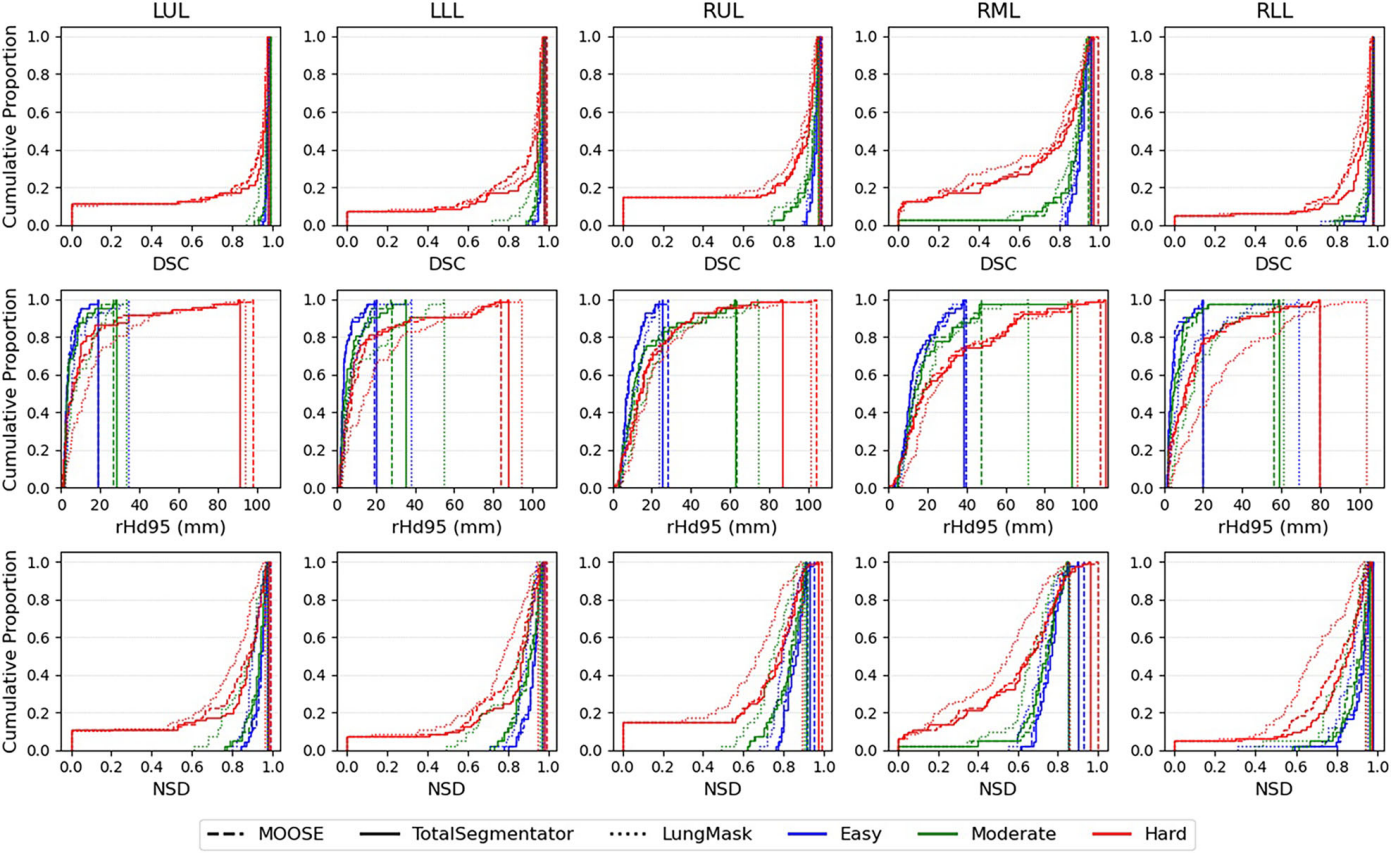
Cumulative distribution functions (proportion of segmented lobes with performance metric less than or equal to the metric value) of DSC, rHd95, and NSD metrics showing the lung lobe segmentation performance of MOOSE, TotalSegmentator, and LungMask across different lobes and difficulty categories (*n* = 164 images; 42 easy, 41 moderate, and 81 hard). DSC, Dice similarity coefficient; LLL, Left lower lobe; LUL, Left upper lobe; NSD, Normalized surface distance; rHd95, 95th percentile Hausdorff distance; RLL, Right lower lobe; RML, Right middle lobe; RUL, Right upper lobe

Figure 2 evaluates the same performance metrics at the lobar level using cumulative distribution functions (CDF), indicating the proportion of lobes (*y*-axis) with a performance metric equal to or less than the corresponding value on the *x*-axis. These results confirm the clear correlation between performance and segmentation difficulty categories, with hard cases consistently displaying lower performance (longer and higher left tails of the CDF for DSC and NSD, and a more gradual rise for rHd95) than moderate cases across all lobes for all metrics.

While MOOSE and TotalSegmentator models generally outperform LungMask when averaged across all lobes, rHd95 results for the RML in hard cases are equally as low as LungMask. This under-segmentation of the RML, caused by difficulty in detecting the right horizontal fissure, in turn leads to RUL over-segmentation. However, because the RUL has a larger surface area, these errors are less impactful and more likely to be excluded as outliers in rHd95 calculations.

NSD (and DSC, similarly) shows consistent underperformance for RML and RUL across all difficulty classes compared to other lobes, which confirms the challenges of segmenting these lobes. However, since these metrics evaluate the overall predicted volume, the impact of RML and RUL segmentation errors is less pronounced compared to rHd95 (Fig. 2).

#### FP prediction

Table 2 summarizes the frequency with which each model incorrectly predicted the presence of a missing lobe (cases with fewer than 5 lobes, *n* = 35). Except for a single scan where LungMask correctly excluded a collapsed LUL, none of the models correctly accommodated any missing lobes.

**Table 2:** The number (percentage) of class false positive predictions for MOOSE, TotalSegmentator, and LungMask across all lobes in cases of missing lobes due to lobectomy and lobe collapse

*n* = 35	False-positive class (%)									
	Lobectomy					Complete lobe collapse				
	RUL (11)	RML (2)	RLL (5)	LUL (6)	LLL (2)	RUL (1)	RML (1)	RLL (0)	LUL (4)	LLL (3)
MOOSE	11 (100%)	2 (100%)	5 (100%)	6 (100%)	2 (100%)	1 (100%)	1 (100%)	_	4 (100%)	3 (100%)
TotalSegmentator	11 (100%)	2 (100%)	5 (100%)	6 (100%)	2 (100%)	1 (100%)	1 (100%)	_	4 (100%)	3 (100%)
LungMask	11 (100%)	2 (100%)	5 (100%)	6 (100%)	2 (100%)	1 (100%)	1 (100%)	_	**3** (**75%)**	3 (100%)

Bold values indicate instances in which a model correctly identified a missing lobe

Column header numbers in parentheses represent the number of images, with each having < 5 lobe segments

*LUL* Left upper lobe, *LLL* Left lower lobe, *RUL* Right upper lobe, *RML* Right middle lobe, *RLL* Right lower lobe

### In-house developed model evaluation

#### Quantitative performance

**Fig. 3:**
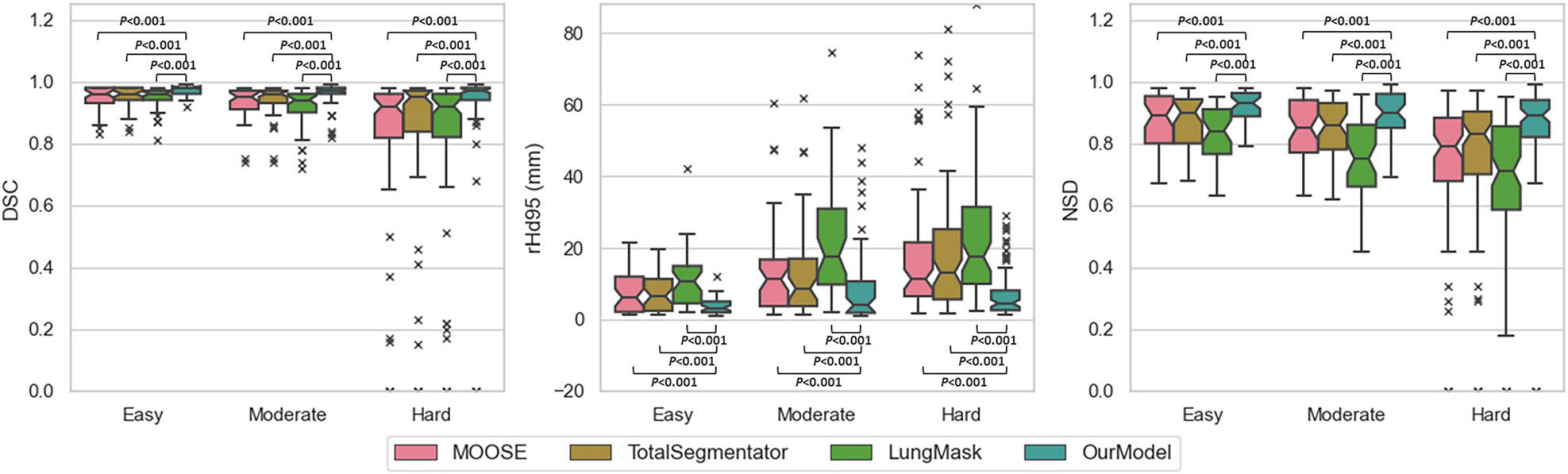
Boxplots displaying the performance of our model compared to the three other segmentation models, based on each evaluation metric across different difficulty categories. From left to right, the boxplots represent the performance evaluated using the DSC, rHd95, and NSD metrics, respectively (*n* = 33 images; 8 easy, 9 moderate, and 16 hard). DSC, Dice similarity coefficient; NSD, Normalized surface distance; rHd95, 95th percentile Hausdorff distance

In evaluating MOOSE, TotalSegmentator, LungMask, and our in-house trained model on the 20% test subset (*n* = 33), our model demonstrated superior performance across all difficulty categories, with the highest median DSC (≥ 0.97), lowest rHd95 (≤ 4.54 mm), and highest NSD (≥ 0.89) across easy, moderate, and hard cases (Table 3). Figure 3 compares the performance of our model with that of the three open-source models on this external test set, showing statistically significantly better performance (*p* < 0.001) for all difficulty groups and performance metrics. Visually, it may appear that rHd95 results for our model for the hard category are better than those for the moderate category, which is contrary to expectations. However, this only relates to the high values (Q3 and outliers). The median rHd95 was smaller but did not reach statistical significance (*p* = 0.782). Nevertheless, these data suggest that performance remains unchanged between moderate and hard cases with our model, an improvement over the other models, which all showed a significant drop in performance in hard cases. These results are also summarized in Table 3.

**Table 3:** Segmentation performance for open-source models and our model on the internal test subset (*n* = 33)

*n* = 33	Easy (8)					Moderate (9)					Hard (16)				
	Mean	SD	Q1	Median	Q3	Mean	SD	Q1	Median	Q3	Mean	SD	Q1	Median	Q3
	**DSC**														
MOOSE	0.95	0.04	0.93	0.96	0.98	0.93	0.05	0.91	0.95	0.97	0.8	0.29	0.82	0.92	0.96
TotalSegmentator	0.95	0.04	0.94	0.96	0.98	0.94	0.05	0.93	0.96	0.97	0.81	0.29	0.84	0.95	0.97
LungMask	0.94	0.04	0.94	0.96	0.97	0.91	0.07	0.9	0.94	0.96	0.8	0.29	0.82	0.92	0.96
Our model	**0.97**	**0.02**	**0.96**	**0.98**	**0.98**	**0.96**	**0.04**	**0.96**	**0.97**	**0.98**	**0.89**	**0.24**	**0.94**	**0.97**	**0.98**
	**rHd95 (mm)**														
MOOSE	7.61	5.83	2.15	6.13	11.99	13.11	12.82	3.73	11.22	16.79	17.26	16.41	6.53	11.3	21.49
TotalSegmentator	7.43	5.35	2.45	6.52	11.35	13.02	13.29	3.76	8.63	17.03	17.65	17.38	5.64	13.13	25.15
LungMask	11.12	7.81	4.54	10.66	15.01	21.67	16.26	9.81	17.77	30.89	22.78	17.99	9.99	17.53	31.37
Our model	**3.69**	**2.28**	**1.99**	**3.2**	**5.05**	**9.73**	**12.24**	**1.88**	**4.03**	**10.69**	**7.24**	**6.89**	**2.6**	**4.54**	**8.14**
	**NSD**														
MOOSE	0.87	0.09	0.8	0.89	0.95	0.84	0.1	0.77	0.85	0.94	0.71	0.27	0.68	0.79	0.88
TotalSegmentator	0.87	0.09	0.8	0.9	0.94	0.84	0.1	0.78	0.86	0.93	0.73	0.27	0.7	0.83	0.9
LungMask	0.83	0.09	0.76	0.84	0.91	0.76	0.13	0.66	0.75	0.86	0.66	0.26	0.58	0.71	0.86
Our model	**0.92**	**0.05**	**0.89**	**0.93**	**0.96**	**0.89**	**0.08**	**0.85**	**0.9**	**0.96**	**0.82**	**0.23**	**0.82**	**0.89**	**0.94**

Results are combined across all lobes for each difficulty class. Bold values represent the highest-performing models in each metric, difficulty class, and model category. Column header numbers in parentheses represent the number of images

Further analysis of only the hard cases with respect to those with (*n* = 7) and without (*n* = 9) missing lung lobes is shown in Fig. S3. Visually, it is evident that DSC and NSD values as small as zero were observed, consistent with the FP prediction of absent lung lobes. rHd95 values, which excluded metrics for missing lobes (as they are undefined), also showed decreased performance (higher values) associated with erroneous lung lobes predicted. This pattern was true for all external models, but not for our model, which had relatively consistent performance even in the absence of lung lobes.

#### FP prediction

**Fig. 4:**
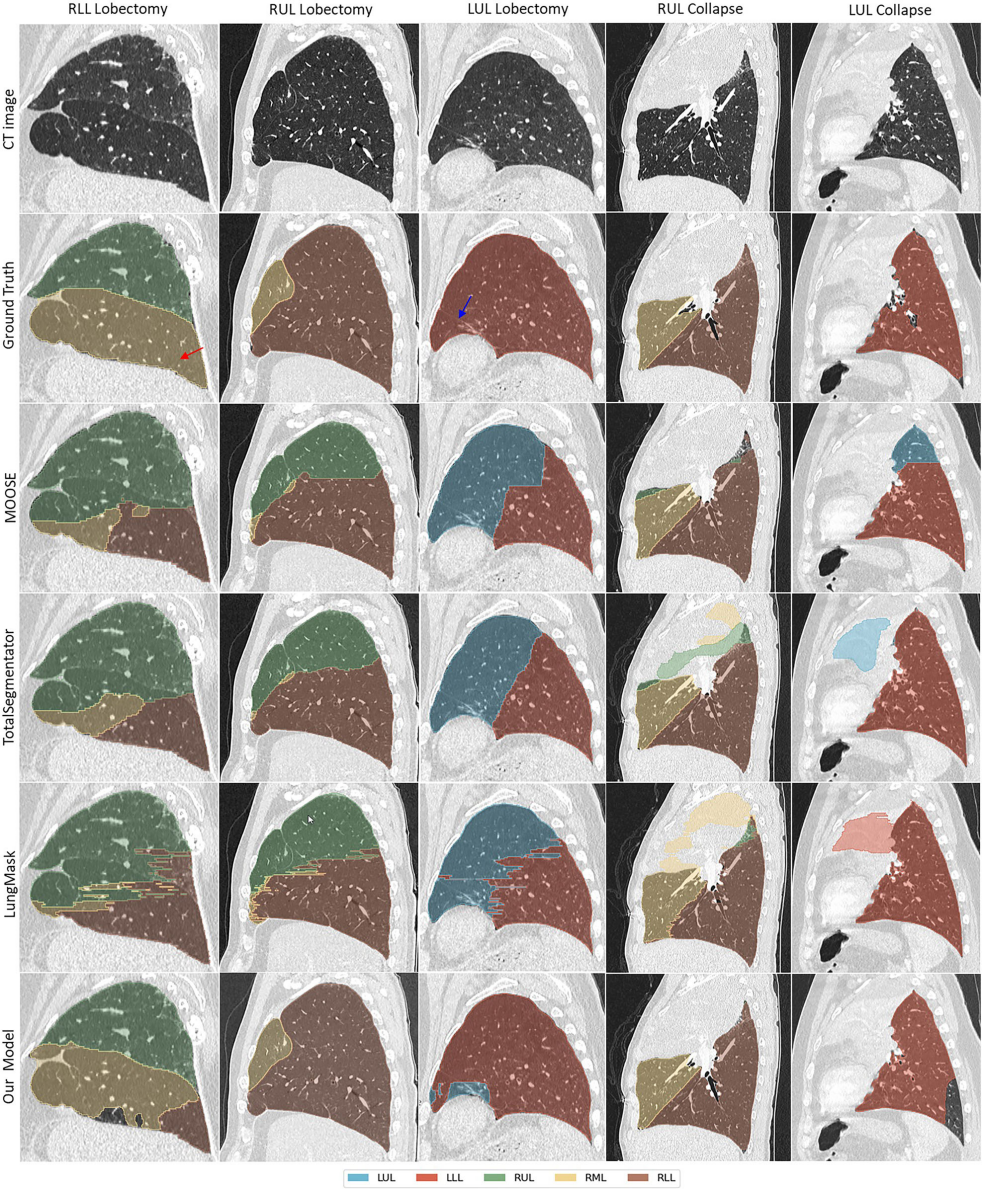
Qualitative results demonstrating the performance of all models in handling various cases with missing lobes. As outlined in Supplemental material (Lung lobe segmentation difficulty classification), any cases with fewer than the typical five lobar annotations were classified under the hard difficulty category. The blue arrow points to the remnant of the left lung’s main fissure following a LUL lobectomy, while the red arrow points to a structure resembling a fissure. LLL, Left lower lobe; LUL, Left upper lobe; RLL, Right lower lobe; RML, Right middle lobe; RUL, Right upper lobe

Due to the limited number of images with missing lobes, our test dataset included only 7 cases: 4 lobectomies (2 RUL, 1 RLL, and 1 LUL) and 3 collapsed lobes (1 RUL, 1 LUL, and 1 LLL). All cases resulted in FPs, except for the collapsed LUL in LungMask and three cases in our model: one RUL lobectomy, collapsed RUL, and collapsed LUL. Although the sample size is small, these results suggest that our model accommodates missing lobes more effectively (3/7) than any of the open-source models. Figure 4 shows five example cases segmented using all four models *versus* the ground truth. Even in instances where our model mistakenly predicted a missing lobe, these segments were much smaller than those of other models (see Supplemental Material: Missing Lobe Cases).

### Performance on the external dataset

#### Quantitative performance

**Fig. 5:**
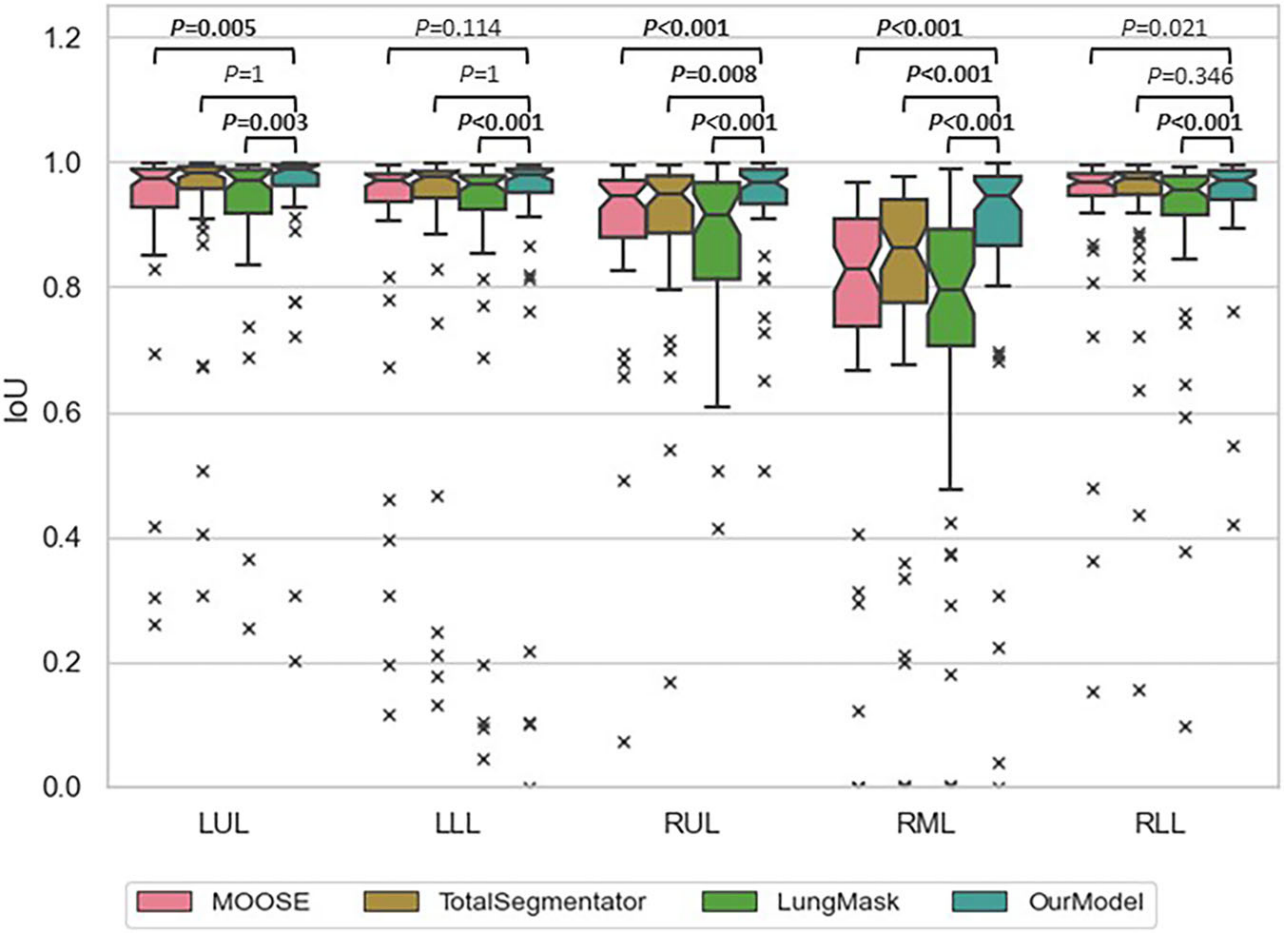
Boxplot of intersection over union (IoU) of all models across all lobes, evaluated on the external dataset (*n* = 55 images). LLL, Left lower lobe; LUL, Left upper lobe; RLL, Right lower lobe; RML, Right middle lobe; RUL, Right upper lobe

Figure 5 compares the performance of our model to that of three open-source models across all lung lobes on the 55 external scans using the IoU metric. Consistent with our internal test set results, our model demonstrated superior generalization to this out-of-sample test dataset, achieving statistically significantly better performance than the other models in predicting RML and RUL (*p* ≤ 0.008) and outperforming LungMask across all lobes (*p* < 0.001). See Supplemental Material (Evaluation of the external dataset).

### Computational considerations

A comparison of the required compute resources and associated image segmentation runtimes is summarized in Table 4.

**Table 4:** Segmentation algorithm required resources and measured runtimes

Model	Chest CT scan (512, 512, 258)			Whole-body CT scan (512, 512, 390)		
	RAM (GB)	VRAM (GB)	Runtime (min:s)	RAM (GB)	VRAM (GB)	Runtime (min:s)
MOOSE	2.13	2.10	1:06	3.89	2.68	2:06
TotalSegmentator*	5.06	5.20	2:36	12.52	12.56	7:54
LungMask	4.59	4.60	6:00	5.22	4.60	3:54
OurModel	4.12	3.08	1:36	4.59	11.16	6:18

* TotalSegmentator does not provide a dedicated model specifically for lung segmentation. As a result, the resources and runtime required for this model are based on the prediction of all organs and anatomical structures present in a chest CT scan

## Discussion

In this work, we sought to evaluate the efficacy of available open-source segmentation software for segmenting the lung lobes in the context of patients referred to the nuclear medicine department for lung lobe function quantification. This population represents a subset of patients with confirmed pulmonary conditions, assessed for presurgical lung lobe function quantification, encompassing diseases and comorbidities such as lung lesions, pneumothorax, pleural effusion, collapsed lobes, emphysema, and fibrotic pulmonary diseases. Based on our segmentation difficulty categorization, all software performed similarly and well when typical lung anatomy was preserved, and fissures were clearly visualized (“easy” cases). However, as anatomical changes and fissure distortion increased from “moderate” to “hard” cases, overall segmentation performance in terms of both fissure delineation and lung tissue segmentation degraded, and performance differed between software.

Another goal of this work was to evaluate whether lung lobe segmentation performance in this challenging population could be improved by developing our own model trained on our collated representative cohort. We used a small, but representative image set and a network architecture similar to that of MOOSE and TotalSegmentator to demonstrate that the performance can indeed be enhanced by incorporating more representative and diverse cases. We confirmed this finding using an external database and independent scoring of the segmentation results from all software. Performance was improved in terms of quality of segmentation and fewer predictions of non-existing lung lobes, *i.e*., fewer FPs.

Regarding the in-house model performance on the internal dataset, as illustrated in Fig. 3 for the rHd95 metric, our model unexpectedly performed better in the hard category than in the moderate category at the upper range of values. This anomaly could be explained due to the exclusion of seven lobes corresponding with cases with missing lobes; all in the hard category (see Performance evaluation on the internal dataset), which may have made the remaining cases less representative. Overall, the remaining hard cases exhibit fewer surface area-related segmentation errors compared to the moderate cases, which predominantly involve lesions. Since the results of other models do not exhibit the same pattern, this highlights the superior performance of our model in handling hard cases, excluding those with missing lobes, compared to the others, while also highlighting its challenges in addressing lesions present in moderate cases. Apart from these findings, our model’s performance continued to reflect task difficulty, achieving its best results with easy cases but progressively declining with increased difficulty.

**Fig. 6:**
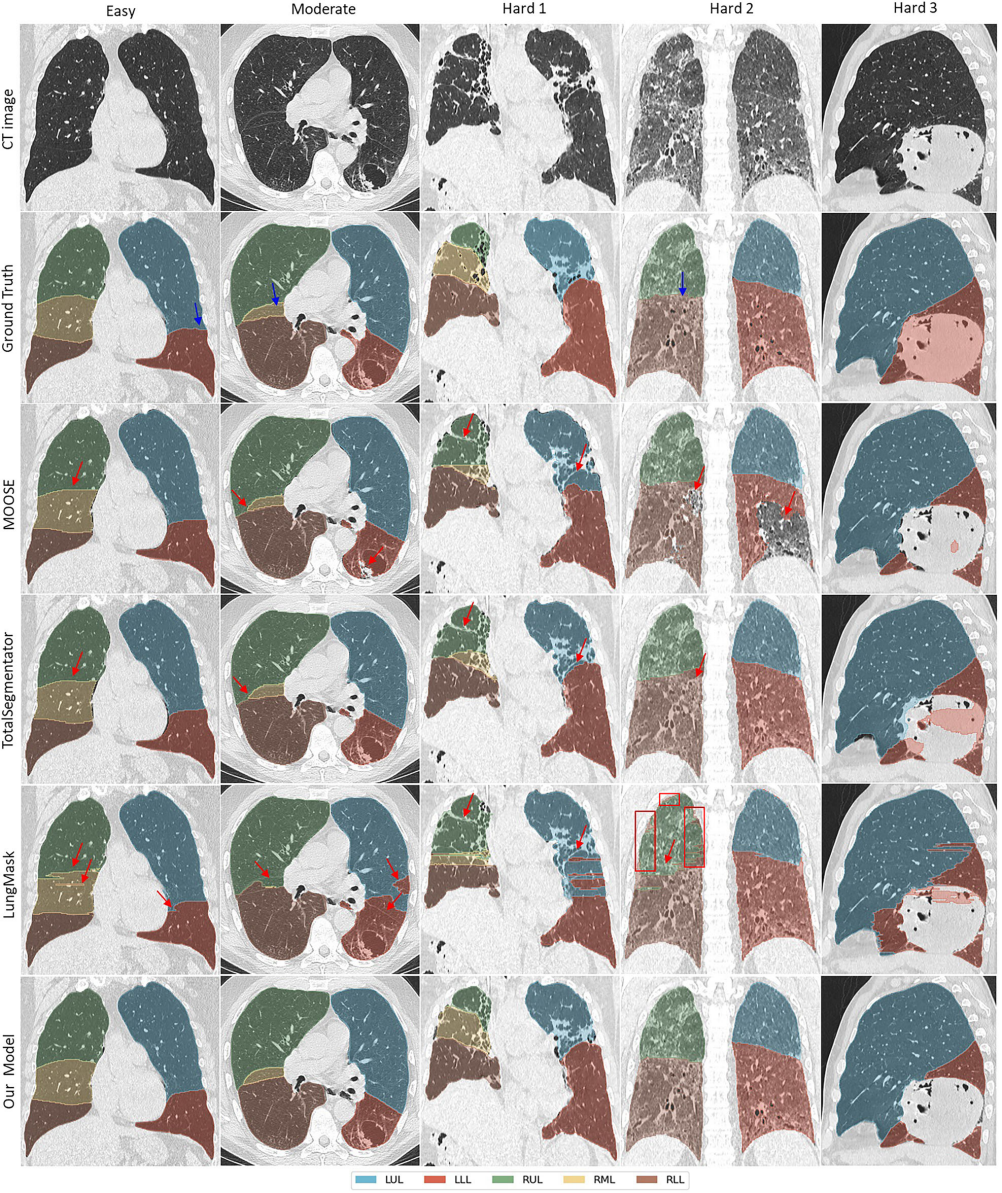
Qualitative results demonstrating the performance of all models across all difficulty categories. Blue arrows highlight ground truth annotation errors related to fissure delineation, while red arrows indicate prediction errors in locating the fissures and the inclusion of lesions and diseased lung regions. Red rectangles highlight areas of further segmentation inaccuracies specific to LungMask. LLL, Left lower lobe; LUL, Left upper lobe; RLL, Right lower lobe; RML, Right middle lobe; RUL, Right upper lobe

Building on the quantitative analysis of the performance of the models, we present the qualitative results across all difficulty categories on our in-sample test set in Fig. 6. The columns, from left to right, represent examples of an easy case, a moderate case, and three hard cases. The easy case has normal anatomy, while the moderate case includes a > 3 cm cystic lesion and mild lung changes. The hard cases feature severe fibrosis, distorted fissures, ground-glass opacities, and large masses. These examples highlight the range of CT scans needing segmentation in clinical practice.

In all these cases, our model consistently outperformed others in lobar delineation and parenchyma segmentation, accurately identifying fissures even in cases with slight test ground truth annotation errors (*e.g*., blue arrows). It successfully classified cystic lesions as part of the lobes, as seen in the moderate case, but excluded opaque lesions, such as the hard 3 mass. MOOSE and TotalSegmentator consistently struggled with right horizontal fissure segmentation, as discussed in the Open-source software evaluation section, especially in hard cases, while LungMask’s 2D approach led to jagged edges and poor segmentation in the presence of pathologies. In the hard 2 case, MOOSE presented the least favorable outcome by excluding large areas of diseased tissue, and LungMask misclassified pleural edges (*e.g.*, red rectangles). TotalSegmentator included more of the hard 3 lesions than others, but this may be a coincidental result of overtraining on typical anatomy rather than accurate modeling of this abnormality, as evidenced by the partial inclusion. Red arrows highlight prediction errors in fissure localization and the inclusion of lesions or diseased lung regions.

Regarding the in-house model performance on the external dataset, our model demonstrated superior overall performance in the LOLA11 challenge dataset in IoU compared to the other models in this work, emphasizing the importance of data variability over sheer dataset quantity in achieving superior model performance, as demonstrated by our model being trained on a much smaller sample size than MOOSE and TotalSegmentator. Our model struggled with missing lungs, where the absent right lung and left lung shift led to FP predictions. However, such cases are rare; none of the 210 patients scanned in our department over six years had a missing lung, with only two showing severe anatomical changes.

As alluded to, to benefit clinical application, training of segmentation models on diverse and representative datasets is essential. This can be achieved through local training of a model, as we have done, which is onerous and not practical to all but a few clinics with strong research support. Alternatively, regulatory-approved commercial entities could develop and deliver such models, which would require their access to datasets through collaboration with clinics. So long as there is still a human-in-the-loop overseeing the segmentation results and manual software is available to correct erroneous segmentations, open-source models may be another avenue to the clinic.

We recognize that the main limitation of this work is potential ambiguity regarding the ground truth segmentation consistency of the images and how it impacts the performance evaluation of the discussed models. We did make a concerted effort to define a segmentation strategy and manually correct semi-automated segmentation results from the employed clinical software. Among the evaluated models, LungMask was the only model trained on a dataset with lesion inclusion for training and evaluation. In contrast, the other models, including ours, were trained on data excluding solid lesions (with CT density ≥ soft tissue). Consequently, to ensure fair comparison, we did not alter our training data (*n* = 131), which aligns with the majority of models evaluated. However, we believe that pulmonary lesions should be included in lung lobe annotations (see Supplemental Material: Annotation Challenges and Considerations), and thus we corrected all test cases (*n* = 33) to include lesions, if present. We acknowledge this approach may have disadvantaged LungMask on the full dataset, but three points mitigate this concern: (1) most lesions were small relative to total lung volume and therefore, had minimal impact on metrics. For context, excluding a spherical lesion of 3 cm in diameter (a common size in our dataset) from a lung lobe with a volume of around 550 cc would remove approximately 14.1 cc, or roughly 2.5% of the lobe’s volume. This would correspond to a reduction in Dice coefficient of only about 1.3%; (2) despite being trained with lesion-inclusive data, LungMask failed to segment large lesions, such as the prominent lesion shown in Fig. 6 (Case hard 3); and (3) it consistently performed significantly worse than other models on the lesion-inclusive test set (*n* = 33), as shown in Table 3 and Fig. 3, and also on the external LOLA11 dataset, suggesting that the performance gap is not attributable to annotation mismatch alone.

While our in-house model demonstrated superior performance in handling cases with missing lobes, we acknowledge that the limited number of such cases in the test set (*n* = 7) restricts our ability to generalize our findings with high confidence. However, the small sample size reflects the clinical rarity of these conditions compared to other pulmonary abnormalities.

We further examined the model’s performance in missing lobe cases and, while some errors occurred in underrepresented scenarios, the model showed encouraging signs of generalization. Anecdotally, it was able to correctly segment a right upper lobe (RUL) collapse case during testing, despite having no examples of RUL collapse in the training set (Fig. 4). This suggests a level of robustness, potentially stemming from exposure to similar anatomical variations during training.

In these cases, simple manual postprocessing can be a practical solution. However, to systematically improve model robustness in these challenging cases, two complementary strategies can be considered: (1) increasing the representation of such variations in the training dataset, and/or (2) incorporating prior expected anatomical or clinical knowledge into the model through tailored loss functions or rule-based post-processing frameworks.

It is important to highlight that overall, all models provided good-quality segmentations for a significant proportion of cases, making them suitable for clinical use in lung function quantification, where precise fissure delineation is not always essential. However, as disease complexity increases and lung anatomy becomes more abnormal, the performance of these models tends to degrade.

In conclusion, lung lobe segmentation on CT images is a uniquely complicated task due to the diversity of disease manifestations, congenital abnormalities, and variations in image acquisition and reconstruction protocols. While state-of-the-art open-source segmentation algorithms that have been trained on thousands of CTs perform well in most cases, they fail to produce adequate results in complex cases that deviate from normal anatomy. The inclusion of representative complex cases in the training of these algorithms can greatly enhance their performance.

## Supplementary information

**Additional file 1: Table S1**. Criteria used to assign a segmentation difficulty category to each chest CT scan. **Fig. S1**. Flowchart illustrating the data collection process, including the total number of cases initially collected, the exclusion criteria applied, and the final dataset used for analysis. **Fig. S2**. Boxplots displaying the performance metrics for open-source models across different difficulty categories for all lobes combined. From left to right, the box plots represent the performance evaluated using the DSC, rHd95, and NSD metrics, respectively. All *p*-values are after a Bonferroni correction factor of 3 was applied (*n* = 129 images; 42 Easy, 41 moderate, and 46 hard, ×5 segments per image). **Fig. S3**. Comparative analysis of segmentation accuracy of hard cases from the internal test set (*n* = 16), specifically those without missing lobes (*n* = 9) and those with missing lobes (*n* = 7), indicating that missing lobes have a disproportionately large impact on segmentation accuracy for all models except our model. **Fig. S4**. Bar chart comparing IoU scores for five lung lobes across 55 cases from the LOLA11 dataset. The chart is divided into two panels: Cases 1−28 and Cases 29−55. Each bar represents the IoU score for a specific lobe (LUL, LLL, RUL, RML, RLL) in each case, with scores ranging from -1.0 to 1.0, where -1.0 indicates excluded evaluations, 0 indicates no overlap, and 1.0 indicates perfect overlap between the model prediction and the ground truth. IoU, Intersection over union; LLL, Left Lower Lobe; LUL, Left Upper Lobe; RML, Right Middle Lobe; RLL, Right Lower Lobe; RUL, Right Upper Lobe. **Fig. S5**. Qualitative results showcasing some of the instances of the LOLA11 challenge dataset where our model successfully performed. Conditions included noisy scans, low-resolution images, emphysematous changes, and lesions ranging from small to moderate in size, both cystic and solid. Red arrows indicate areas where the model failed to make accurate predictions. LLL, Left lower lobe; LOLA11, LObe and Lung Analysis 2011; LUL, Left upper lobe; RLL, Right lower lobe; RML, Right middle lobe; RUL, Right upper lobe. **Fig. S6**. Qualitative results showcasing instances where our model struggled to perform accurately. The conditions and pathologies presented in these cases include disease- and treatment-related missing lobes (cases 44, 45, and 48), disease-related volume loss (cases 06, 20, 31, and 52), large lesions (case 31), and emphysematous changes (case 28). Red arrows indicate areas where the model failed to make accurate predictions, including misidentified fissures, exclusion of high-density parenchymal and pleural regions, and false positive class predictions. LLL, Left lower lobe; LUL, Left upper lobe; RLL, Right lower lobe; RML, Right middle lobe; RUL, Right upper lobe. **Fig. S7**. Two cases from the LOLA11 challenge where no overlap between our model’s prediction and ground truth annotations was recorded, with an IoU of 0 reported for the RML in case 21, and the LLL in case 52. In case 52, the red arrow highlights the ground truth region labeled as LLL, while the blue arrow indicates the location of the left oblique fissure. This visual interpretation calls into doubt the accuracy of ground truth segmentation and/or annotations in the LOLA11 competition. IoU, Intersection over union; LLL, Left lower lobe; LOLA11, LObe and Lung Analysis 2011; LUL, Left upper lobe; RLL, Right lower lobe; RML, Right middle lobe; RUL, Right upper lobe. **Fig. S8**. Example slices of LOLA11 challenge cases with missing right lung (case 44), missing left lung (case 45), and missing RML (case 48), demonstrating varying degrees of segmentation accuracy by the four models evaluated. The red arrow indicates a structure resembling the right horizontal fissure. LLL, Left lower lobe; LOLA11, LObe and Lung Analysis 2011; LUL, Left upper lobe; RLL, Right lower lobe; RML, Right middle lobe; RUL, Right upper lobe.

## Data Availability

A large portion of the data, including the CT and co-registered lung perfusion SPECT scans with corresponding lung lobes and trachea annotations, is available at 10.5281/zenodo.12690802. Note that these annotations have been modified prior to submission and may undergo further updates. The remaining data, with cleaned annotations, will be made available in the future. Original study annotations are available from the corresponding author upon reasonable request. Authors using the shared data must cite both the data repository and this manuscript.

## Abbreviations

CT: Computed tomography
DSC: Dice similarity coefficient
FP: False positive
LLL: Left lower lobe
LOLA11: LObe and lung analysis 2011
LUL: Left upper lobe
MOOSE: Multi-organ objective segmentation
NSD: Normalized surface distance
rHd95: 95th Percentile Hausdorff distance
RLL: Right lower lobe
RML: Right middle lobe
RUL: Right upper lobe
SPECT: Single-photon emission computed tomography
