# Supplementary material for: Lung lobe segmentation: performance of open-source MOOSE, TotalSegmentator, and LungMask models compared to a local in-house model
Source: Eur Radiol Exp. 2025 Sep 4;9:86. doi: 10.1186/s41747-025-00623-9 (PMC12411369; doi:10.1186/s41747-025-00623-9)
Supplement: Supplementary file 1 — Additional file 1: Table S1. Criteria used to assign a segmentation difficulty category to each chest CT scan. Fig. S1. Flowchart illustrating the data collection process, including the total number of cases initially collected, the exclusion criteria applied, and the final dataset used for analysis. Fig. S2. Boxplots displaying the performance metrics for open-source models across different difficulty categories for all lobes combined. From left to right, the box plots represent the performance evaluated using the DSC, rHd95, and NSD metrics, respectively. All p-values are after a Bonferroni correction factor of 3 was applied (n = 129 images; 42 Easy, 41 moderate, and 46 hard, ×5 segments per image). Fig. S3. Comparative analysis of segmentation accuracy of hard cases from the internal test set (n = 16), specifically those without missing lobes (n = 9) and those with missing lobes (n = 7), indicating that missing lobes have a disproportionately large impact on segmentation accuracy for all models except our model. Fig. S4. Bar chart comparing IoU scores for five lung lobes across 55 cases from the LOLA11 dataset. The chart is divided into two panels: Cases 1−28 and Cases 29−55. Each bar represents the IoU score for a specific lobe (LUL, LLL, RUL, RML, RLL) in each case, with scores ranging from -1.0 to 1.0, where -1.0 indicates excluded evaluations, 0 indicates no overlap, and 1.0 indicates perfect overlap between the model prediction and the ground truth. IoU, Intersection over union; LLL, Left Lower Lobe; LUL, Left Upper Lobe; RML, Right Middle Lobe; RLL, Right Lower Lobe; RUL, Right Upper Lobe. Fig. S5. Qualitative results showcasing some of the instances of the LOLA11 challenge dataset where our model successfully performed. Conditions included noisy scans, low-resolution images, emphysematous changes, and lesions ranging from small to moderate in size, both cystic and solid. Red arrows indicate areas where the model failed to make accurate predictions. LLL, Le [file 41747_2025_623_MOESM1_ESM.pdf]

Lung lobe segmentation: performance of open-source MOOSE, TotalSegmentator, and LungMask models compared to a local in-house model

ELECTRONIC SUPPLEMENTARY MATERIAL

Open-Source software tools

Multi-Organ Objective Segmentation (MOOSE)

The MOOSE segmentation algorithm, developed by Sundar et al. [18] processes both CT and ^{18}F-FDG PET scans, using PET data (if provided) solely to measure the regional tracer uptake within the segmented regions. It offers 14 segmentation models, including one for lung lobe segmentation, trained on the AutoPET dataset [26] and an additional external lung cancer dataset, comprising 1243 whole-body PET/CT scans in total. The model uses the nnU-Net model architecture. MOOSE is publicly available.

TotalSegmentator

Isensee et al. [19] developed TotalSegmentator, a medical image segmentation tool that offers segmentations for 117 organs and major anatomical structures on CT images. The segmentation model is also built on the nnU-Net architecture and was trained on 1,228 whole-body CT scans, with varied pathologies. The segmentation model and the training data with the corresponding annotations are all publicly available [20].

Johof Lung Segmentation (LungMask)

Hofmanninger et al. [21] developed a two-phase study for lung lobe segmentation, herein referred to as LungMask. In phase one, they used a 2D U-Net model trained on a diverse dataset of 231 CT scans with various lung pathologies. The 2D results were combined to create a 3D segmentation. In phase two, they trained another 2D U-Net for lung lobe segmentation using a subset of the Lung Tissue Research Consortium (LTRC) [27] dataset which predominantly comprises lung scans from patients with chronic obstructive pulmonary disease (COPD) and interstitial lung disease (ILD). While the first dataset is not publicly available, the LTRC dataset can be accessed upon request.

Task difficulty assignment

In our study, we deliberately categorized all our cases into three difficulty levels of Easy, moderate, and hard to segment, intending to assess the performance of algorithms under significantly different pathological and anatomical conditions. However, assigning difficulty was inherently subjective, relying on the assessor's understanding of how algorithms perform under these varied scenarios. To address this challenge, we defined key image attributes that were known to impact task performance. By consistently applying these criteria, we aimed to make the classification process more reproducible. The correlative drop in performance with task difficulty is a confirmation of our task difficulty assignment.

To select accurate criteria influencing the segmentation performance of algorithms, we visually assessed each scan and segmentation results from both conventional (i.e. Hybrid3D™) and machine learning-based methods (i.e. previous iterations of in-house software), alongside radiology reports on disease presence. Key criteria identified included abnormal lung tissue density, history of lobectomy, atelectasis, anatomical changes due to therapy or pathology, and presence of lesions. Based on these factors, we scored each CT scan on a 3-point scale (Easy, moderate, hard) in terms of a segmentation model’s expected ability to accurately detect and delineate lung fissures and to correctly segment the lobes, ensuring inclusion of all tissues, both healthy and diseased, within the corresponding segmented region of the lung parenchyma.

In Table S1, we have summarized our category assignments. Satisfaction of just one criterion of a category is sufficient to assign that category to a scan. For each scan, we first assessed the criteria for the hard category and if none of the hard criteria were met, we then considered the moderate criteria, and finally, the Easy criteria. For instance, if there was a history of lobectomy or lobe atelectasis, regardless of the presence of other factors, that scan was categorized as hard. Thus, the final image score assigned corresponds to the hardest scoring criteria that were met.

Table S1 Criteria used to assign a segmentation difficulty category to each chest CT scan.

Image score	Scoring criteria
**Hard**	• Any lung that has undergone lobectomy<br>• Any lung that had undergone (near) complete lobe collapse.<br>• Severe diffused hyper- or hypo-attenuation within the lung parenchyma (> -700 or < -900 HU) affecting fissure location and perceptibility.<br>• Radiology reported severe cases of pneumothorax or hydropneumothorax, or their presence causing compression to the lung lobes resulting in a reduction of lobe(s) volume by more than 50% of normal volume.<br>• Lesions larger than 3 cm in at least two dimensions resulting in fissure displacement.<br>• Lesions larger than 3 cm in at least two dimensions occupying more than 50% of lobe volume.<br>• Severe volume loss/reduction (more than 50%), either locally or globally, regardless of the underlying cause (*e.g.*, wedge resection, local atelectasis, fibrosis, or pleural effusion).
**Moderate**	• Focal hyper- or hypo-attenuation within the lung parenchyma (>-700 or <-900 HU) leading to ambiguity regarding lung boundary delineation.<br>• Radiology reported moderate pneumothorax, hydropneumothorax cases.<br>• Pleural effusion cases causing 30-50% lobe reduction.<br>• Lesions larger than 1 cm but smaller than 3 cm in size, adjacent to, but not displacing, the fissures or pleura.<br>• Any large lesion not meeting hard category criteria.<br>• Mild to moderate volume loss (30-50%), either localized or affecting the entire lung.
**Easy**	• Any case that does not fall into the hard or moderate categories.

Evaluation of the external dataset

Fig. further details the IoU score for all five lung lobes across 55 cases as reported by the LOLA11 competition portal for our model. As shown, an IoU value of -1 was recorded for certain lobes in scans 44 and 45, representing pathologies that made it impossible to differentiate lobes within a lung, and case 48, representing a missing lobe. Lobes marked with a score of -1.0 were excluded from all evaluations in the challenge, as per the organizers' decision.

To further illustrate our model's strengths, Fig. demonstrates our model's strengths across various challenging cases in the LOLA11 dataset, excelling with cystic lesions, severe bronchiectasis, moderate lesions, severe emphysema, noisy data, and low-resolution images.

Fig. S6, in contrast, highlights cases where our model performed poorly. including severe volume loss (cases 06, 20, 31, 52), bullous emphysema (case 28), large lesions (case 31), missing lobes (cases 44, 45, 48), and treatment-induced changes (case 52). Cases 44, 48, and 52 showed the most significant misclassifications.

Limitations of the external dataset

We identified two key limitations in the LOLA11 dataset. First, ground truth annotations for the RML (case 21) and LLL (case 52) appear inconsistent (see Fig. S7). In case 21, all models, including ours, received an IoU of 0 for the RML. This is unexpected because if the RML were truly absent or unidentifiable, the LOLA11 protocol should have assigned a score of -1, as it did for other missing lobes (cases 44, 45, and 48). This raises concerns about whether the lobe was mislabeled or incorrectly annotated. If it were present, we would expect at least some overlap between predictions and the ground truth, but all models scored 0, suggesting a potential labeling error. Likewise, in case 52, our model received an IoU of 0 for the LLL, despite predicting a region that clearly matched the anatomical lobe. The LOLA11 annotation included only a small area (red arrow), which our model didn't capture. A consulting physician confirmed that our prediction aligned with the true LLL and followed the left oblique fissure (blue arrow). Notably, our model's prediction for the adjacent LUL scored 0.99, ruling out simple misclassification. In contrast, MOOSE, TotalSegmentator, and LungMask received non-zero IoUs (0.40, 0.21, and 0.04) by including both the small annotation and the anatomically correct region, but this likely reflects a reliance on learned spatial patterns rather than accurate anatomical reasoning and still resulted in segmentation errors. Since LOLA11 is closed for competition, disputing scores was not possible.

Second, LOLA11 assigns a score of -1.0 for lobes that cannot be differentiated due to severe pathology or are missing (Cases 44, 45, 48), excluding them from evaluation. This design limits assessing how well models handle missing lobes, an important factor in overall performance. While our model did predict lobes that were absent in some cases, it outperformed MOOSE and TotalSegmentator, which overfitted on learned spatial patterns (Fig. S8, case 45). Instead, it demonstrated sensitivity to anatomical cues and was able to follow structures resembling fissures (red arrow) when present, rather than blindly segmenting based on expected lobe locations (Fig. S8, case 48). Nevertheless, we acknowledge that our model led to the worst result in the missing right lung case (Fig. S8, case 44), where it incorrectly predicted right lung lobes in the left lung. Despite gross

segmentation errors in case 44, all IoU values were relatively high (>0.87), attributable to the large LUL volume in this case.

Missing lobe cases

Our curated dataset included 35 cases with fewer than 5 typical lung lobes, 9 due to lobar collapse (3 in the test set and 6 in the training set) and 26 due to lobectomy (4 in the test set and 22 in the training set). While lobectomy causes significant anatomical changes, collapse is reversible with minimal changes to the lung anatomy. Therefore, to evaluate the models' performance in each scenario, we separately analyzed false positive predictions for lobectomy and collapsed cases.

Cases with missing lobes are rare in clinical practice, so we made sure to include at least two examples of each missing lobe scenario in our test set, especially for challenging lobectomies. However, some variations, such as RML lobectomy, LLL lobectomy, RML collapse, RLL collapse, or cases involving multiple excised or collapsed lobes, were not represented. Therefore, the model's performance in those specific scenarios remains unassessed. However, it is noteworthy that these variations are also rarer to encounter in clinical practice, and their underrepresentation is not unique to our dataset but also likely absent in the training sets of the evaluated open-source models.

Even with this limited training dataset of a diverse presentation, the model performance in missing lobes was greatly enhanced. This gives us confidence that with more examples of lobectomy and collapsed lungs, the model can be further enhanced to accommodate missing lobes.

Annotation challenges and considerations

Segmentation of lung CT faces unique challenges, especially in annotating lesions and extra-pulmonary spaces.

We aimed to include solid masses (CT density equal to or greater than soft tissue) in lung lobe segmentations despite their lack of physiological function, for three key reasons: (1) Lesions are anatomically part of the lung parenchyma, reflecting anatomical reality. (2) Lung tissue surrounding lesions often change (e.g., inflammation, fibrosis), making precise manual exclusion tedious and inconsistent, potentially impacting model training and validation. (3) In SPECT-based lobar function assessments, SPECT activity may overlap lesion areas due to its low resolution (~10–15 mm). Therefore, including lesions ensures accurate lobar activity quantification for applications like pre-operative planning.

This decision to include lesions in the parenchymal space may not be ubiquitous across impacting training of external models and performance metrics reported by LOLA11. MOOSE

and TotalSegmentator were not explicitly trained to segment (include) lesions, unlike LungMask, which was trained with lesions as part of the ground truth segmentations.

However, given that most lesions in our dataset were small relative to total lung volume, their inclusion or exclusion had a negligible impact on the overall performance of models.

Extra-pulmonary abnormalities like pleural effusion and pneumothorax were included in LungMask's ground truth annotations but excluded from our lobe annotations, as they do not represent functional lung tissue. LungMask's performance may have been affected in such cases, but due to their limited occurrence in our dataset, this had minimal impact on overall model performance.

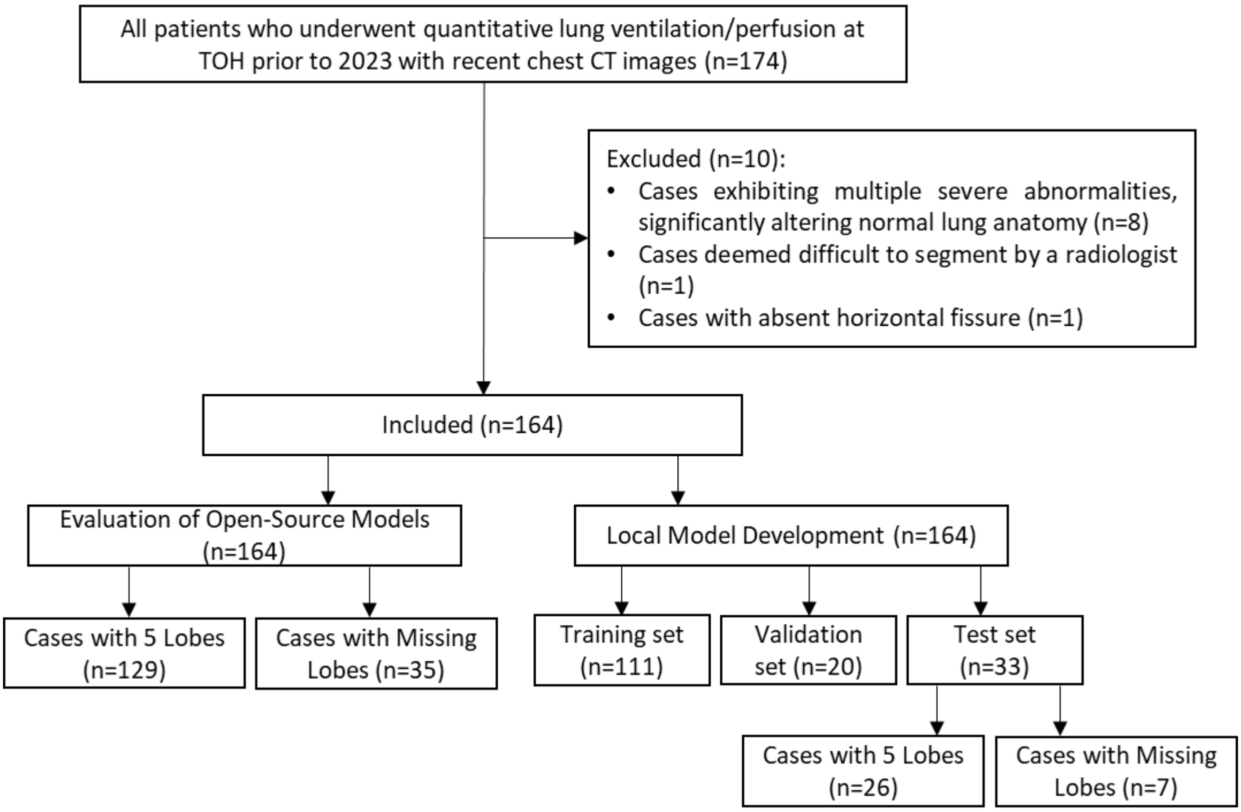

Fig. S1 Flowchart illustrating the data collection process, including the total number of cases initially collected, the exclusion criteria applied, and the final dataset used for analysis.

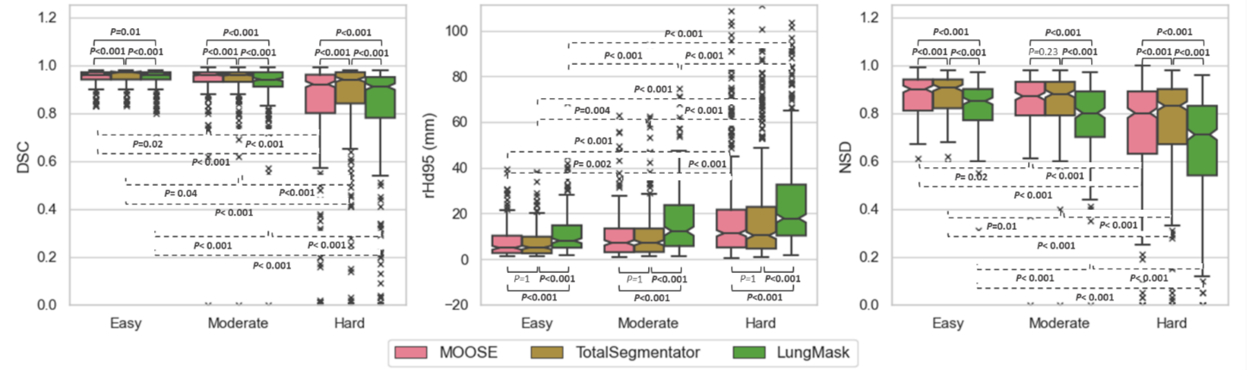

Fig. S2 Boxplots displaying the performance metrics for open-source models across different difficulty categories for all lobes combined. From left to right, the boxplots represent the performance evaluated using the DSC, rHd95, and NSD metrics, respectively. All p-values are after a Bonferroni correction factor of 3 was applied (n=129 images; 42 Easy, 41 moderate, and 46 hard, × 5 segments per image).

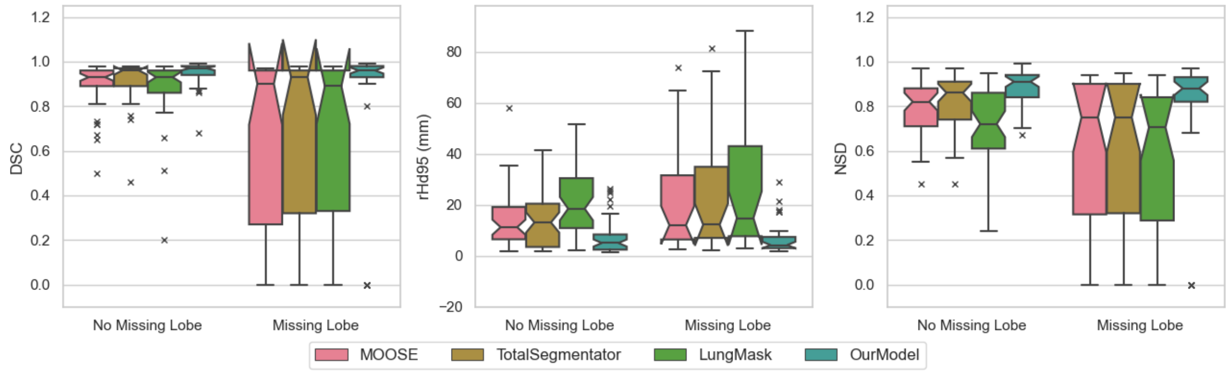

Fig. S3 Comparative analysis of segmentation accuracy of hard cases from the internal test set (*n* = 16), specifically those without missing lobes (n=9) and those with missing lobes (*n* = 7), indicating that missing lobes have a disproportionately large impact on segmentation accuracy for all models except our model.

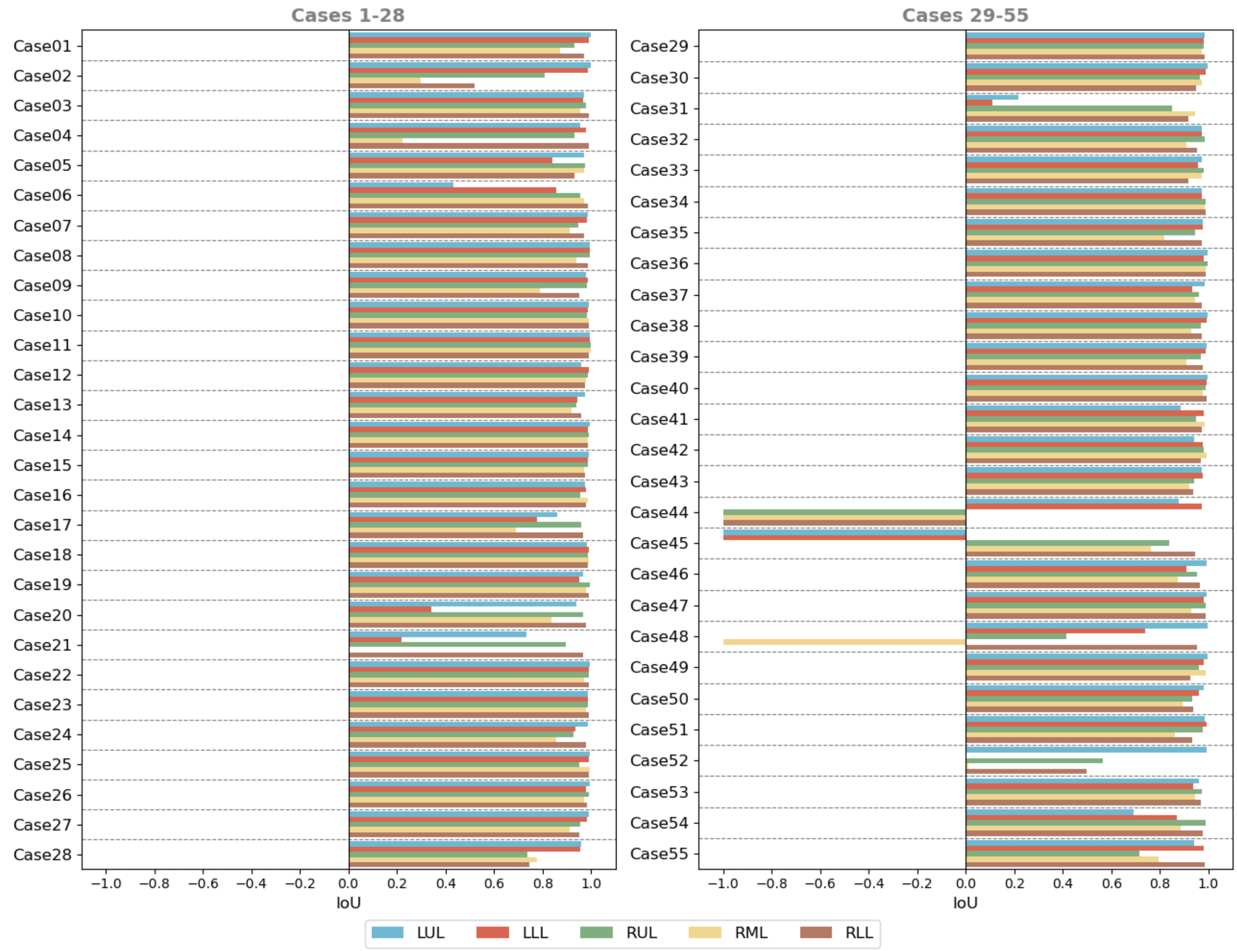

Fig. S4 Bar chart comparing IoU scores for five lung lobes across 55 cases from the LOLA11 dataset. The chart is divided into two panels: Cases 1−28 and Cases 29−55. Each bar represents the IoU score for a specific lobe (LUL, LLL, RUL, RML, RLL) in each case, with scores ranging from -1.0 to 1.0, where -1.0 indicates excluded evaluations, 0 indicates no overlap, and 1.0 indicates perfect overlap between the model prediction and the ground truth. *IoU* Intersection over union, LLL: Left Lower Lobe, LUL: Left Upper Lobe, RML: Right Middle Lobe, RLL: Right Lower Lobe, RUL: Right Upper Lobe.

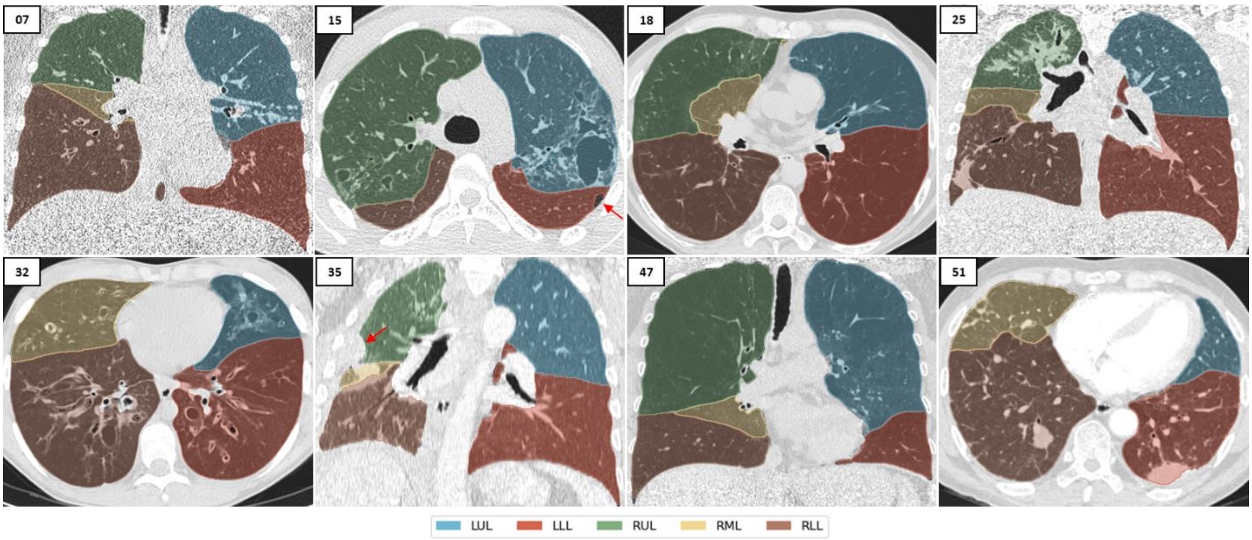

Fig. S5 Qualitative results showcasing some of the instances of the LOLA11 challenge dataset where our model successfully performed. Conditions included noisy scans, low-resolution images, emphysematous changes, and lesions ranging from small to moderate in size, both cystic and solid. Red arrows indicate areas where the model failed to make accurate predictions. *LLL* Left lower lobe, *LOLA11* LObe and Lung Analysis 2011, *LUL* Left upper lobe, *RLL* Right lower lobe, *RML* Right middle lobe, *RUL* Right upper lobe.

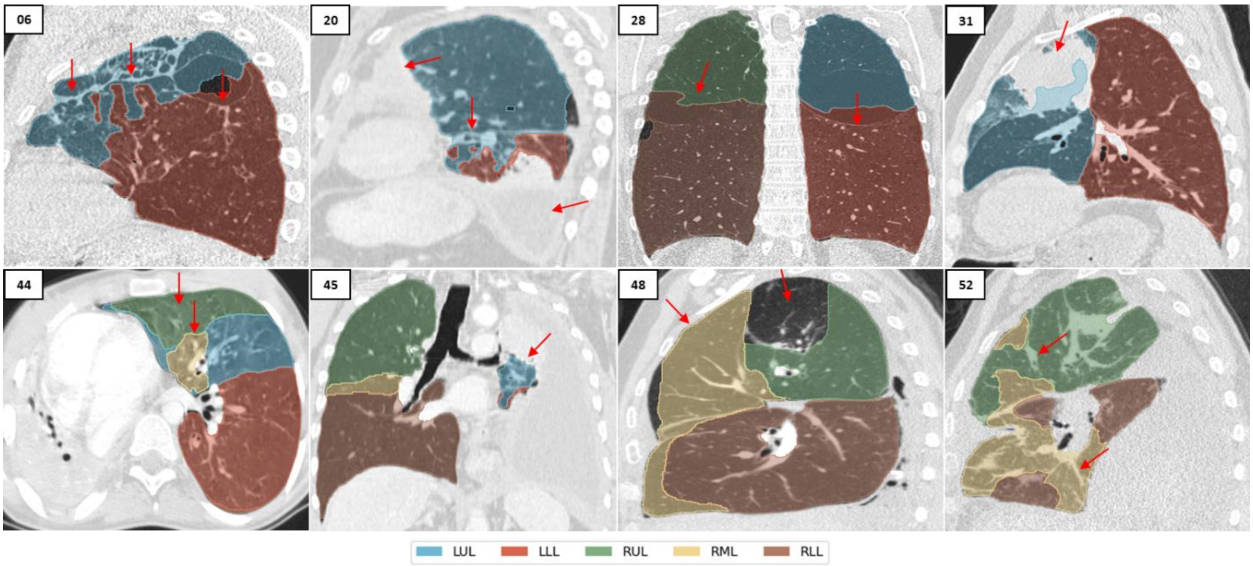

Fig. S6 Qualitative results showcasing instances where our model struggled to perform accurately. The conditions and pathologies presented in these cases include disease- and treatment-related missing lobes (cases 44, 45, and 48), disease-related volume loss (cases 06, 20, 31, and 52), large lesions (case 31), and emphysematous changes (case 28). Red arrows indicate areas where the model failed to make accurate predictions, including misidentified fissures, exclusion of high-density parenchymal and pleural regions, and false positive class predictions. *LLL* Left lower lobe, *LUL* Left upper lobe, *RLL* Right lower lobe, *RML* Right middle lobe, *RUL* Right upper lobe.

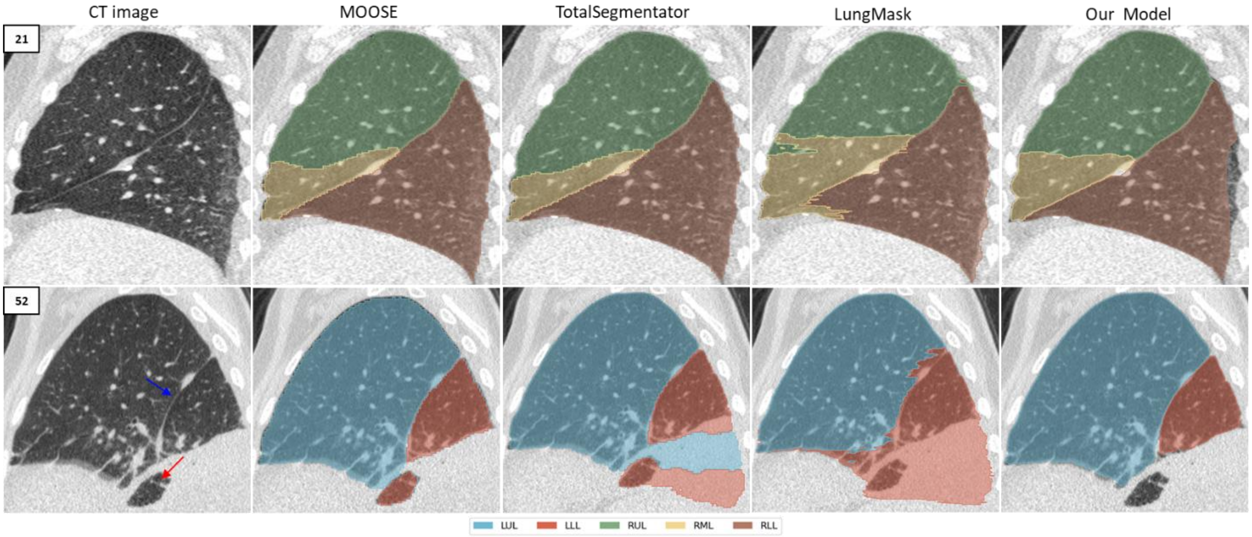

Fig. S7 Two cases from the LOLA11 challenge where no overlap between our model's prediction and ground truth annotations was recorded, with an IoU of 0 reported for the RML in case 21, and the LLL in case 52. In case 52, the red arrow highlights the ground truth region labeled as LLL, while the blue arrow indicates the location of the left oblique fissure. This visual interpretation calls into doubt the accuracy of ground truth segmentation and/or annotations in the LOLA11 competition. *IoU* Intersection over union, *LLL* Left lower lobe, *LOLA11* LObe and Lung Analysis 2011, *LUL* Left upper lobe, *RLL* Right lower lobe, *RML* Right middle lobe, *RUL* Right upper lobe.

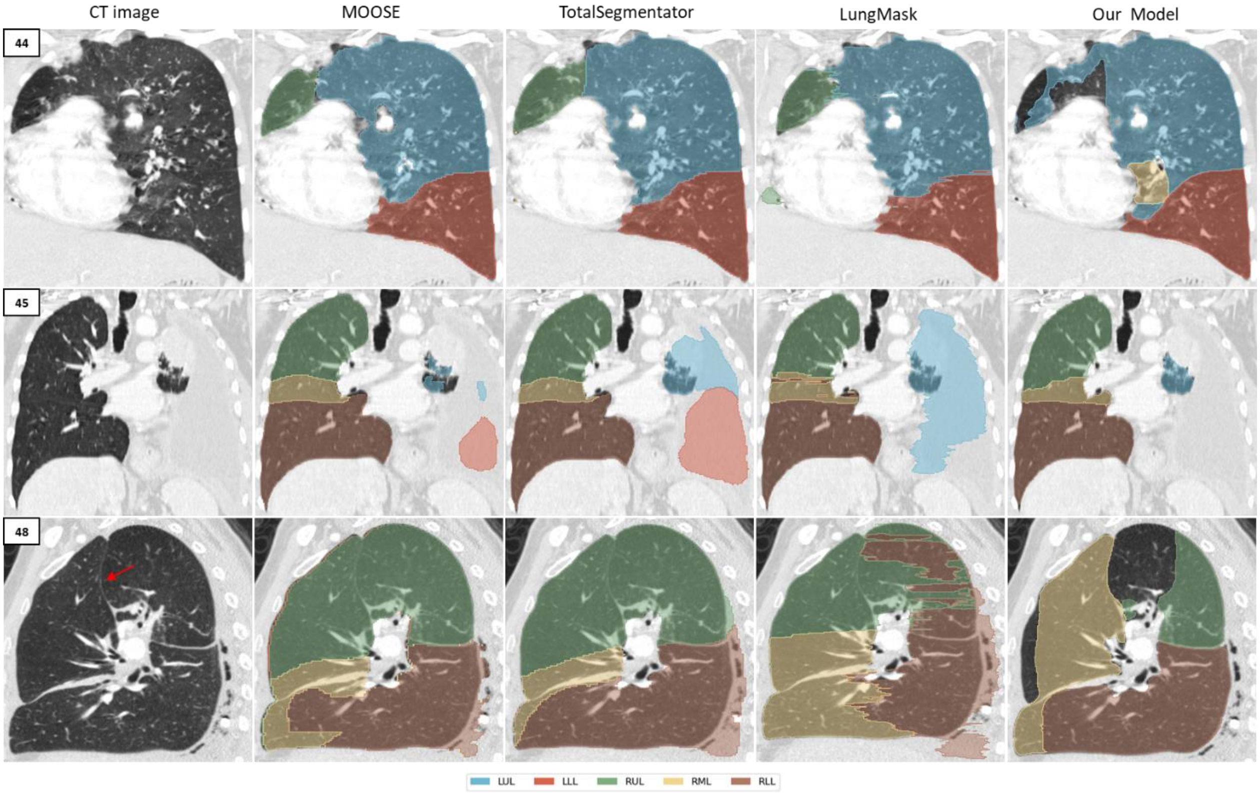

Fig. S8 Example slices of LOLA11 challenge cases with missing right lung (case 44), missing left lung (case 45), and missing RML (case 48), demonstrating varying degrees of segmentation accuracy by the four models evaluated. The red arrow indicates a structure resembling the right horizontal fissure. *LLL* Left lower lobe, *LOLA11* LObe and Lung Analysis 2011, *LUL* Left upper lobe, *RLL* Right lower lobe, *RML* Right middle lobe, *RUL* Right upper lobe.
